# Predictors of young maternal age at first birth among women of reproductive age in Nigeria

**DOI:** 10.1371/journal.pone.0279404

**Published:** 2023-01-13

**Authors:** Obasanjo Afolabi Bolarinwa, Bright Opoku Ahinkorah, Abdul-Aziz Seidu, Aliu Mohammed, Fortune Benjamin Effiong, John Elvis Hagan, Olusesan Ayodeji Makinde

**Affiliations:** 1 Department of Public Health Medicine, School of Nursing and Public Health, University of KwaZulu-Natal, Durban, South Africa; 2 Institute for Advanced Studies in the Humanities, University of Edinburgh, Edinburgh, United Kingdom; 3 School of Public Health, University of Technology Sydney, Sydney, Australia; 4 College of Public Health, Medical and Veterinary Sciences, James Cook University, Townsville, Australia; 5 Centre for Gender and Advocacy, Takoradi Technical University, Takoradi, Ghana; 6 Department of Health, Physical Education and Recreation, University of Cape Coast, Cape Coast, Ghana; 7 Department of Clinical Chemistry and Immunology, Faculty of Medical Laboratory Science, University of Calabar, Calabar, Nigeria; 8 Neurocognition and Action-Biomechanics-Research Group, Faculty of Psychology and Sport Sciences, Bielefeld University, Bielefeld, Germany; 9 Viable Knowledge Masters, Abuja, Nigeria; 10 Viable Helpers Development Organization, Abuja, Nigeria; University of Mississippi Medical Center, UNITED STATES

## Abstract

**Background:**

Adverse obstetric outcomes have been commonly associated with early childbearing in many low-and middle-income countries. Despite this evidence, scholarly information on early childbearing in the sub-Saharan African region, especially Nigeria, is limited. This study examines the predictors of young maternal age at first birth among women of reproductive age in Nigeria using multi-level analysis.

**Methods:**

Data from the most recent Nigeria Demographic and Health Survey conducted in 2018 were analyzed. A total of 29,949 women of reproductive age (15–49 years) were considered for the study. Descriptive statistics using weighted percentage and chi-square test of independence (χ^2^) were first used to describe the variables of interest. This procedure was followed by a multilevel analysis of factors associated with young maternal age at first birth in Nigeria at p<0.05 level of significance.

**Results:**

Approximately 36.80% of the sample population had their first birth before the age of 18. Mothers residing in the North-East region [aOR = 1.26; 95% (CI = 1.13–1.42)] and practicing Islam [aOR = 1.17; 95% (CI = 1.05–1.29] were more likely to have their first birth before the age of 18 than those in the North-Central region and those practicing Christianity. Living in communities with medium literacy level [aOR = 0.90; 95% (CI = 0.82–0.99)] and high literacy level [aOR = 0.71; 95% (CI = 0.62–0.81)], being within richest wealth index [aOR = 0.61; 95% (CI = 0.53–0.71)] and being Yoruba [aOR = 0.46; 95% (CI = 0.39–0.56)] were associated with lower odds of young maternal age at first birth.

**Conclusion:**

More than one-third of women of reproductive age in Nigeria had given birth to their first child before 18 years. Thus, there is a need for the Nigerian government and other stakeholders, including Non-Governmental Organisations and Civil Society Organisations to formulate and implement policy interventions targeted at reducing early childbearing among women of reproductive age in Nigeria.

## 1. Background

Pregnancy and childbirth-related complications are the leading cause of death among girls aged 15–19 years worldwide, with most of these deaths occurring in low-and middle-income countries (LMICs), especially in sub-Saharan Africa (SSA) [1, 2]. Thus, early childbearing (below the age of 18) remains a major public health challenge in SSA, including Nigeria [3, 4], and this is because of its association with increased maternal and infant morbidity and mortality [1, 5].

Available evidence suggests that young maternal births have an increased risk for adverse maternal health outcomes, including haemorrhage, obstructed labour [6], puerperal endometritis, eclampsia and systemic infections [2]. Besides these, early motherhood has also been associated with adverse child health outcomes such as preterm delivery, low birthweight [2, 3], malnutrition and poor development [1].

Young mothers are more likely to give birth to children with mental retardation, academic difficulties and behavioural problems [5, 7–9]. Besides, young motherhood has also been associated with a vicious cycle of poverty, because most women who give birth early are likely to be less educated and unemployed. Therefore, they may not have the needed resources to support their children’s development [1]. Furthermore, adolescent mothers may also experience high school dropout rates and suffer from low self-esteem [10, 11].

According to the 2013 Nigeria Demographic and Health Survey (NDHS), about 23% of women between the ages of 15 and 19 years have already started childbearing [12].Although the proportion of young mothers aged 15–19 years declined between 2013 and 2018 from 23% to 19% [13], the proportion of young mothers within this age group is still high [14, 15], and this has been reported in previous studies conducted in the country [1, 12].On one hand, social issues and structural factors such as age at first marriage, rape, child labour, lower socio-economic status, place of residence and parental educational attainments promote early childbirths [4, 16–20]. On the other hand, studies have suggested a higher educational level as one of Nigeria’s most important protectors of early motherhood [17, 21, 22]. Mothers with a higher educational level are more likely to better understand the risks or complications associated with early pregnancy and childbirth [17, 23]. Additionally, a higher level of education is also associated with improved decision-making capacity of women [24] and increased use of contraceptives [25], thereby delaying and/ or spacing pregnancies.

Despite the importance of age at first birth on maternal and child health outcomes, and the concerted effort by government and non-governmental agencies to promote series of interventions in order to reduce the high prevalence of early childbirths in Nigeria [26, 27], These interventions couldn’t account for some salient predictors of maternal age at first birth among women of reproductive age in Nigeria [26, 27].

Besides, all the previous studies that examined predictors of maternal age at first birth in Nigeria used datasets more than 10 years old [28, 29]. To the best of our knowledge, no study in Nigeria has been done using data from the recent 2018 NDHS dataset. This study, therefore, examines the predictors of maternal age at first birth among women of reproductive age in Nigeria using multi-level analysis. Findings from this study will help develop interventions to reduce early motherhood in the country while prioritizing the mother and child’s life and safety.

## 2. Materials and methods

### 2.1. Study setting and data source

#### 2.1.1. Study setting

The study setting was Nigeria. Nigeria is the most populous country in Africa, occupying the 10^th^ position in the global population chart with an estimated total population of 221 million people [30]. Nigeria’s total fertility rate is currently 5.5 children per woman of reproductive age, with crude birth rates and unintended pregnancies of 42 births per 1,000 live births and 59 pregnancies per 1000 population, respectively [31–33].

#### 2.1.2. Data source

The 2018 NDHS was used for this study. NDHS was chosen because of its accuracy and completeness in capturing data relating to maternal and child health indicators in Nigeria every five years [32]. For this study, the individual recode file was used, and this file contains the responses of women aged 15–49 in Nigeria. In selecting the sample for the 2018 NDHS survey, a stratified dual-stage sampling approach was employed. In line with the study criteria, eligible women were administered questionnaires to elicit information on maternal and child health, including other sexual and reproductive health variables. Details of the sampling approach have been described elsewhere[13, 32]. This study included all the women between 15–49 years with at least a child and had complete cases on all the variables of interest (n = 29,949).

### 2.2. Study variables

#### 2.2.1. Outcome variable

Age at first childbirth was the outcome variable in this study. This variable was derived from the question, “how old were you when you first gave birth?” The responses to this question were in single years. Age at first birth was categorised into more than or equal to 18 years (adult maternal age at first birth) and less than 18 years (young maternal age at first birth). The outcome variable was categorised in line with previous studies [22, 34, 35].

#### 2.2.2. Explanatory variables

Twelve explanatory variables were considered in this study and were grouped into individual-level variables and household/community-level variables. These variables were determined from previous studies and based on their availability in the dataset [36–38].

#### 2.2.3. Individual-level factors

The individual-level factors were education, marital status, occupation, media exposure, religion, and ethnicity. The level of education was coded as ‘no education’ ‘primary’, and ‘secondary/higher’. Marital status was recoded into ‘married’, and ‘unmarried. ‘Working’ and ‘not working’ were the categories for working status. Frequency of reading newspaper/magazine, listening to the radio and watching television were re-categorised as media exposure. Those who didn’t engage in any of these were coded ‘no’ while those exposed to at least one were coded ‘yes’. Religion was coded as Christianity, Islam, and Traditionalist/Others. Ethnicity was coded as Hausa, Yoruba, Igbo, and Others.

#### 2.2.4. Household/Community-level factors

The household/community level variables were the place of residence, region, wealth quintile, sex of the household head, community literacy level and community socio-economic status. Place of residence was coded as ‘urban’ and rural, wealth index and region of respondents as household/community level variables were based on their categorisation in the Demographic Health Survey (DHS) [32]. In DHS, the wealth quintile is computed using Principal Component Analysis (PCA) into 5 categories [39]. The Sex of the household head was coded as ‘male’ and ‘female’. Community literacy level and community socio-economic status were created from the level of education of women and wealth quintile at the community level using PCA. Each of these were categorized into low, medium, and high.

### 2.3. Statistical analyses

The descriptive analysis table displays the prevalence of maternal age at first birth, and the chi-square test of independence (χ^2^) to show the association between maternal age at first birth and the explanatory variables. The multilevel logistic regression model (MLRM) was used to examine the association between the individual and household/community factors and maternal age at first birth. The Stata command “melogit” was used in fitting these models. A 2-level model for binary responses was specified, reporting maternal age at first birth below the age of 18 or not for mothers at level 1 (Individual) and at level 2 (Household/community). Four models were constructed altogether. The first model was the empty model/null model (Model 0), which is the model that shows the variance in the outcome variable (maternal age at first birth) attributed to the clustering of primary sampling units (PSUs). This model has no explanatory variable. The second model contained only the individual-level factors (Model I), while Model 3 contained the household/community-level factors (Model II). The final model was the complete model (Model III) that simultaneously controlled individual and household/community factors.

The MLRM consists of fixed and random effects [40, 41]. The fixed effects (measures of association) showed results of the association between the explanatory variables and the outcome variable (maternal age at first birth) and were reported as adjusted odds ratios (aOR) with their 95% confidence intervals (CIs), while the random effects (measures of variations) were assessed using Intra-Cluster Correlation (ICC) [42, 43]. The LR test was used to check for model adequacy. Akaike’s Information Criterion (AIC) and Bayesian Information Criteria (BIC) were used to measure how well the different models fit the data. The sample weight (v005/1,000,000) was applied to correct for over-and under-sampling, while the SVY command was used to account for the complex survey design and generalizability of the findings. All the statistical analysis was performed with Stata 17.0 (StataCorp, College Station, TX, USA).

### 2.4. Ethical approval

Ethical permissions were not required for this study since NDHS datasets are publicly available at https://dhsprogram.com/what-we-do/survey/survey-display-528.cfm and can be used upon request. The Institutions that commissioned, funded or managed the surveys were responsible for ethical procedures. ICF International and National Population Commission (NPopC) approved the NDHS survey in line with the United States Department of Health and Human Services regulations to protect human subjects.

## 3. Results

### 3.1. Descriptive results on the distribution of maternal age at first birth among women in Nigeria by the explanatory variables

Table 1 provides a summary of the distribution of maternal age at first birth among women in Nigeria by the explanatory variables. The overall prevalence of young maternal age at first birth was 36.80%. At the individual level, women with no education (54.51%), unemployed (45.75%), those practising Islam (48.03%), mothers whose ethnicity were Hausa (54.73%), and unexposed to mass media (50.69%) had a higher chance of first birth before 18 years. There were also variations in the prevalence of maternal age at first birth across the various household/community factors. Mothers residing in the rural area (46.09%), those residing in the North-West region (53.44%), mothers in the poorest wealth quintile (54.24%), those in male-headed households (38.27%), mothers residing in communities with low literacy level (55.24%), and those residing in communities with low socioeconomic status (49.52%) had a higher prevalence of young maternal first birth. All the individual and household/community factors showed significant associations with maternal age first birth.

**Table 1:** Distribution of maternal age at first birth by explanatory variables.

Variables	Frequency (n)	Percentage (%)	Maternal age at first birth (%)		p-values
Individual-level variables			18 years and above	Below 18 years	
**Educational level**					p<0.001
No Education	12,635	42.19	45.49	54.51	
Primary	5,062	16.90	59.38	40.62	
Secondary & above	12,252	40.91	83.06	16.94	
**Currently working**					p<0.001
Not working	8,195	27.36	54.25	45.75	
Working	21,754	72.64	66.58	33.42	
**Marital status**					p<0.001
Unmarried	3,729	12.45	69.11	30.89	
Married	26,220	87.55	62.36	37.64	
**Religion**					p<0.001
Christianity	12,859	42.94	78.05	21.95	
Islam	16,909	56.46	51.97	48.03	
Traditionalist & others	181	0.60	58.33	41.67	
**Ethnicity**					p<0.001
Hausa	11,677	38.99	45.27	54.73	
Yoruba	4,431	14.80	85.21	14.79	
Igbo	4,169	13.92	83.95	16.05	
Others	9,672	32.29	65.83	34.17	
**Media exposure**					p<0.001
No	10,483	35.00	49.31	50.69	
Yes	19,466	65.00	70.68	29.32	
**Household/community level variables**					
**Place of residence**					p<0.001
Urban	12,690	27.33	75.84	24.16	
Rural	17,259	72.67	53.91	46.09	
**Region**					p<0.001
North Central	4,167	13.91	65.34	34.66	
North East	4,891	16.33	50.02	49.98	
North West	9,365	31.27	46.56	53.44	
South East	3,223	10.76	82.74	17.26	
South South	3,247	10.84	76.07	23.93	
South West	5,056	16.88	84.32	15.68	
**Wealth index**					p<0.001
Poorest	5,891	19.67	45.76	54.24	
Poorer	6,241	20.84	50.00	50.00	
Middle	5,976	19.96	61.44	38.56	
Richer	6,036	20.15	72.49	27.51	
Richest	5,805	19.38	87.26	12.74	
**Sex of household head**					p<0.001
Male	25,904	86.49	61.73	38.27	
Female	4,045	13.51	72.62	27.38	
**Community literacy level**					p<0.001
Low	10,180	33.99	44.76	55.24	
Medium	9,428	31.48	61.95	38.05	
High	10,341	34.53	82.51	17.49	
**Community Socioeconomic status**					p<0.001
Low	15,541	51.89	50.48	49.52	
Medium	3,3810	11.29	64.36	35.64	
High	11,027	36.82	80.78	19.22	
**Nigeria**	**n = 29,949**	**100**	**63.20**	**36.80**	

Weighted NDHS, 2018

NB: p-values derived from chi-square test of independence

### 3.2. Fixed effects on measures of association between explanatory variables and maternal age at first birth among women in Nigeria

At the individual-level factors, the likelihood of having first birth below the age of 18 was lower among women who had secondary education & above [aOR = 0.46; 95% (CI = 0.42–0.50)], mothers who were married [aOR = 0.78; 95% (CI = 0.71–0.85)], and women who were from Yoruba ethnic group [aOR = 0.46; 95% (CI = 0.39–0.56)] compared to mothers who were not educated, those who were unmarried, and those from Hausa ethnic group. On the other hand, mothers practicing Islam [aOR = 1.17; 95% (CI = 1.05–1.29)] were more likely to have their first birth below the age of 18 compared to those practicing Christianity.

At the household/community factors, mothers within the richest wealth index [aOR = 0.61; 95% (CI = 0.53–0.71)], those whose household heads were female [aOR = 0.89; 95% (CI = 0.81–0.98)], and those residing in a community with high literacy level [aOR = 0.71; 95% (CI = 0.62–0.81)] were less likely to have their first birth below 18 years compared to mothers within the poorest wealth index, those with a male as household head, and those residing in communities with low literacy level. While mothers residing in North Eastern region [aOR = 1.26; 95% (CI = 1.13–1.42)] were more likely to have their first birth below age 18 compared to those residing in North Central region (Table 2).

**Table 2:** Multilevel logistic regression models for individual and household/community predictors of maternal age at first birth.

Variables	Model 0	Model I	Model II	Model III
		aOR [95% CI]	aOR [95% CI]	aOR [95% CI]
**Fixed effects results**				
**Individual-level variables**				
**Educational level**				
No Education		RC		RC
Primary		0.93[0.85–1.00]		1.01[0.92–1.09]
Secondary & above		0.37***[0.34–0.40]		0.46***[0.42–0.50]
**Currently working**				
Not working		RC		RC
Working		0.97[0.91–1.03]		0.97[0.91–1.03]
**Marital status**				
Unmarried		RC		RC
Married		0.81*** [0.74–0.88]		0.78***[0.71–0.85]
**Religion**				
Christianity		RC		RC
Islam		1.28***[1.17–1.40]		1.17**[1.05–1.29]
Traditionalist & others		1.37*[1.03–1.81]		1.39*[1.05–1.84]
**Ethnicity**				
Hausa		RC		RC
Yoruba		0.31***[0.27–0.35]		0.46***[0.39–0.56]
Igbo		0.36***[0.31–0.41]		0.44***[0.35–0.55]
Others		0.72***[0.66–0.79]		0.78***[0.71–0.86]
**Media exposure**				
No		RC		RC
Yes		0.88***[0.83–0.94]		0.97 [0.91–1.04]
**Household/community level variables**				
**Place of residence**				
Urban			RC	RC
Rural			1.02[0.93–1.12]	1.05[0.96–1.15]
**Region**				
North Central			RC	RC
North East			1.61***[1.44–1.81]	1.26***[1.13–1.42]
North West			1.82***[1.63–2.03]	1.24**[1.09–1.40]
South East			0.56***[0.49–0.65]	1.06[0.83–1.35]
South South			1.01[0.89–1.15]	1.11[0.97–1.27]
South West			0.62***[0.54–0.71]	0.92[0.78–1.09]
**Wealth index**				
Poorest			RC	RC
Poorer			0.96[0.88–1.05]	1.00[0.92–1.09]
Middle			0.88**[0.79–0.97]	0.98[0.89–1.08]
Richer			0.77***[0.68–0.86]	0.96 [0.85–1.08]
Richest			0.41***[0.36–0.48]	0.61***[0.53–0.71]
**Sex of household head**				
Male			RC	RC
Female			0.95[0.88–1.03]	0.89*[0.81–0.98]
**Community literacy level**				
Low			RC	RC
Medium			0.73***[0.66–0.81]	0.90*[0.82–0.99]
High			0.46***[0.40–0.52]	0.71***[0.62–0.81]
**Community socioeconomic status**				
Low			RC	RC
Medium			1.05[0.93–1.18]	0.99 [0.88–1.12]
High			0.98[0.87–1.11]	0.89 [0.79–1.01]
**Random effects results**				
PSU Variance (95% CI)	0.93[0.84–1.04]	0.18[0.14–0.21]	0.17[0.14–0.21]	0.14[0.11–0.17]
ICC	0.22	0.05	0.04	0.04
LR Test	χ2 = 32854.90, p<0.001	χ2 = 248.49, p<0.001	χ2 = 258.32, p<0.001	χ2 = 169.99, p<0.001
Wald χ2	Reference	2464.16***	2090.38***	2781.62***
**Model fitness**				
Log-likelihood	-18338.06	-17385.71	-17592.73	-17253.03
AIC	36680.11	34795.42	35219.45	34560.05
BIC	36696.73	34895.13	35360.70	34784.39
Number of clusters	1389	1389	1389	1389

Weighted NDHS, 2018

Exponentiated coefficients; 95% confidence intervals in brackets; AOR = adjusted Odds Ratios; CI = Confidence Interval; RC = Reference Category.

*p< 0.05

**p< 0.01

***p< 0.001.

AIC = Akaike’s Information Criterion; BIC = Schwarz’s Bayesian Information Criteria; PSU = Primary Sampling Unit; ICC = Intra-Class Correlation; LR Test = Likelihood ratio Test.

Model 0 is the null model, a baseline model without any determinant variable.

Model I is adjusted for individual-level variables (Educational level, currently working, marital status, religion, ethnicity and media exposure).

Model II is adjusted for household/community level variables (Place of residence, region, wealth index and sex of household head).

Model III is the final model adjusted for individual and household/community level variables

### 3.3. Random effects on measures of variations associated with maternal age at first birth among mothers in Nigeria

As shown below in Table 2, the empty model depicted a substantial variation in the likelihood of young maternal age at first birth in Nigeria across the PSUs clustering (σ2 = 0.93; 95%CI = 0.84–1.04). The empty model (Model 0) indicated that 22% of young maternal age variation at first birth in Nigeria was attributed to the variation between-cluster characteristics, i.e., (ICC = 0.22). The variation between-cluster decreased to 5% in Model I, representing only the individual level model (Model I). At the community level, in the only model, which is (Model II), the ICC declined to 4%, while the same 4% ICC was maintained in the complete model (Model III), with both the individual and household/community models. The Model III, which is the complete model with both the selected individual and household/community factors, was chosen to predict the eventuality or occurrence of young maternal age at first birth among mothers in Nigeria.

## 4. Discussion

This study examined the predictors of maternal age at first childbirth among women of reproductive age in Nigeria. The findings show that about 36.80% of women had their first childbirth before the age of 18. Furthermore, the study showed that maternal age at first childbirth was significantly associated with education, marital status, religion, ethnicity, region of residence, wealth index, sex of household head, and community literacy level.

The proportion of women who had their first childbirth below the age of 18 (36.80%) in the current study is lower than the 46.7% and 53.6% previously reported by Neal, Channon [44] in SSA and Wegbom, Wokoma [45] in Nigeria respectively. The difference in the proportion of women who had their first childbirths before 18 years (during adolescence) in the current study compared to the previous studies could be attributed to the different periods in which the studies were conducted, and the variation in the sample sizes across the studies. Whereas both Neal, Channon [44] and Wegbom, Wokoma [45] used the 2013 NDHS, the current study was based on the 2018 NDHS.

Interestingly, our finding perhaps affirms a decreasing trend of early first childbirths in Nigeria. For instance, Neal and Channon’s [44] study on trend analysis of adolescent first births in SSA found that the percentage of women who had their first childbirths below the age of 18 in Nigeria was 53.6%, 42.9% and 46.7% based on the 1990, 2008 and 2013 NDHSs respectively. This trend is quite worrying because there has been a persistent effort to reduce adolescent childbirths in SSA, and much decline is expected than what is currently observed considering the investments and promotions that have been made [46–48]; in the same vein, considering the negative effect of the phenomenon on maternal and child health outcomes [2, 49–51]. Thus, there is a need for concerted efforts to minimise adolescent motherhood in Nigeria in order to attain SDG 3.7 by 2030 [4, 52].

This study showed a significant reduction in the odds of having first childbirth under18 years among women with higher education, those employed and those within the richest wealth index. Previous studies also reported that factors such as higher educational attainment [53–56], being employed [53–55], and belonging to a rich household [54, 56], decrease the likelihood of having first childbirth during adolescence. Perhaps, women with higher education, those employed and those from rich households tend to be more empowered and, therefore, can make the right reproductive health decisions, including when to begin motherhood. For instance, Seidu and Ahinkorah [24] reported that education, wealth index and employment are significantly associated with women’s decision-making capacity and empowerment. Additionally, women with higher education, those employed, and those from rich households are more likely to have easy access to and use contraceptives [50], increasing their likelihood of delaying childbirth.

The lower likelihood of having first childbirth below the age of 18 among educated women and women from rich households could also be explained by the fact that women from rich households are more likely to spend more years in school in pursuit of higher education. Increased years of schooling reduces the chance of early marriage and early sexual debut, both associated with increased adolescent pregnancies and childbirths [56, 57]. Besides, a higher level of education also increases the odds of getting employed, which tends to delay first childbirth among women [53, 55]. Arguably, educated women are more likely to live in urban communities and neighbourhoods with high literacy rates compared to women who were not educated. Findings from this study suggest that women who were residents of communities with high literacy levels had lower odds of getting pregnant before age 18 compared to those in communities with low literacy levels. Living in communities with high literacy levels increases women’s exposure to contraceptive use [25].

In this study, women who were residents of the North Central region had higher odds of first childbirth before age 18 compared to those in the South East and South West regions. Similar findings were made by Ayo, Adeniyi [58], who reported that adolescents’ first childbirth was high in Nigeria’s Northern regions relative to the Southern regions. The variations in age at first childbirth between the Northern and Southern regions of the country could be attributed to the socio-economic, cultural, and religious differences between the two regions.

Socio-economically, women in Northern Nigeria experience high poverty and unemployment rates and low educational levels [59], which increases their risk of giving birth during adolescence [55, 56]. For example, Kyari and Ayodele [60] reported that the high poverty levels in Northern Nigeria tend to portray young girls as an economic burden and therefore marrying them to wealthy men becomes an attractive venture for most parents and guardians. Additionally, the cultural practices of the dominant ethnic groups in Northern Nigeria (Hausa/Fulani) coupled with the dominance of Islamic religious practices tend to promote early marriage [54, 59, 60] and thereby increase the risk of adolescent childbirths in the Northern part of the country [60, 61].

This study also revealed that unmarried women had higher odds of having their first childbirth before 18 years compared to married women. This finding contradicts the findings of previous studies in Nigeria [54]and elsewhere [62], which reported that most adolescent childbirths occur within marriages. These variations could be explained by the differences in the timings of the studies and geographical locations. Perhaps, the high tolerance for premarital sex and the increasing delay of marriages in most SSA countries, including Nigeria [57], could explain the high level of early childbirth among unmarried women. As suggested by the UN [62], early first childbirth tends to remain low in countries where the majority of childbirths are expected to occur within the context of marriages. Thus, in Northern Africa, for instance, the low rate of premarital sex had contributed to the high age at first childbirth [5, 57].

Women residing in female-headed households had a lower likelihood of having their first childbirth before attaining 18 years. This finding contradicts the findings of previous studies in West Africa [61], South Africa [63] and Mexico [64], where being a member of a female-headed household was associated with an increased risk of early childbearing among women. Perhaps, the significant improvement of women’s decision-making capacity and empowerment in Nigeria over the past two decades [65] might have accounted for the observed differences. For example, a recent study reported that residing in a female-headed household increases the odds of using contraceptives among adolescent girls in SSA [25].

### 4.1. Strengths and limitations

This study’s major strength lies in using the most recent nationally representative dataset to investigate maternal age predictors at first childbirth in Nigeria. This enhances the generalisability of the study findings to women of reproductive age in Nigeria. Additionally, because DHS data are collected by well-trained enumerators, the validity of the dataset used in the analysis is high, strengthening the validity of the study findings. Despite the aforementioned strengths, there are some limitations inherent in the study that needs to be acknowledged. The age at first birth was self-reported, which makes it prone to recall desirability bias, and DHS datasets are cross-sectional and cannot be used to make causal inferences.

### 4.2. Policy and public health implications

The prevalence of first childbirths below 18 years is high among women of reproductive age in Nigeria. The phenomenon is largely influenced by factors such as educational status, employment status, media exposure, North-East geographical location, wealth index, marital status, sex of household head and community literacy level. Thus, policymakers and implementers need to recognize and address these factors to minimize women’s vulnerability to early childbirth and its related negative consequences on maternal and child health outcomes. Increasing women’s access to education, employment, and wealth, as well as media exposure and support schemes for women household heads, could significantly improve their decision-making capacity and empowerment. Empowered women can independently make the right reproductive health decisions, including when to begin parenthood, contraceptives, and other family planning methods. Special attention must be given to the women in Northern Nigeria, especially regarding education and poverty alleviation strategies such as entrepreneurial opportunities for adolescent girls. Considering the current threat to girl-child education in Northern Nigeria due to insurgency groups like Boko Haram; there is a need for targeted interventions to improve access and the safety of girls at school. Furthermore, there is a need to enact the gender and equal opportunities law in Nigeria, which has suffered repeated rejections by the legislature [66].

## 5. Conclusions

The study concluded that there is a high prevalence of first childbirths below the age of 18 years among women of reproductive age in Nigeria, and the predictors associated with it include educational level, marital status, religion, ethnicity, the region of residence, wealth index, sex of household head, and community literacy level. There is a need for the Nigerian government and other stakeholders, including Non-Governmental Organisations and Civil Society Organisations to formulate and implement policy interventions targeted at reducing the prevalence of early childbearing among women of reproductive age in Nigeria. Such interventions could improve women’s empowerment through better access to girl-child education, and poverty reduction, especially in the Northern part of the country. Implementation of these strategies would not only improve maternal and child health outcomes in Nigeria but also hasten the attainment of SDG 3.7, which seeks to improve sexual and reproductive health among women.

## Abbreviations

NDHS: Nigeria Demographic and Health Survey
DHS: Demographic Health Survey
LMICs: low-and middle-income countries
PCA: Principal Component Analysis
MLRM: Multilevel logistic regression model
AOR: Adjusted odds ratios
CI: Confidence Interval
ICC: Intra-Cluster Correlation
AIC: Akaike’s Information Criterion
BIC: Bayesian Information Criteria
NPopC: National Population Commission
UN: United Nations
